# Charge Carrier Transport and Localized States in Graphite-like Amorphous Carbon Films at Room Temperatures

**DOI:** 10.3390/ma18173977

**Published:** 2025-08-25

**Authors:** Vyacheslav A. Moshnikov, Ekaterina N. Muratova, Igor A. Vrublevsky, Alexandr I. Maximov, Viktor B. Bessonov, Stepan E. Parfenovich, Alexandr K. Tuchkovsky, Dmitry A. Kozodaev

**Affiliations:** 1 Microelectronics Department, Saint Petersburg Electrotechnical University “LETI”, Professora Popova St., 5, 197022 Saint Petersburg, Russia; vamoshnikov@mail.ru (V.A.M.);; 2 Micro and Nanoelectronics Department, Belarusian State University of Informatics and Radioelectronics, Brovki St., 6, 220013 Minsk, Belarus; vrublevsky@bsuir.edu.by (I.A.V.);; 3 NT-MDT BV, Hoenderparkweg 96 b, 7335 Apeldoorn, The Netherlands

**Keywords:** photovoltaic cell, graphite-like amorphous carbon, transport layer, Raman spectra, I–V characteristic, hopping electrotransport mechanism

## Abstract

The conductivity of direct and alternating current for graphite-like amorphous carbon films after annealing in vacuum at a temperature of 700 °C was studied. The I–V characteristics of such films are symmetrical. The I–V curve in logarithmic coordinates demonstrated the presence of two linear sections. A study of the frequency dependences of structures with a thin graphite-like amorphous carbon film showed a sharp increase in capacitance at low frequencies and a decrease in the high-frequency region. The increase in capacitance in the low-frequency region is explained by the Maxwell–Wagner polarization, which is observed in inhomogeneous dielectrics with conducting inclusions. The results of temperature measurements of resistance showed that at room temperatures, there is a mechanism of conduction of electrons with a variable jump length along localized states lying in a narrow energy band near the Fermi level. At the same time, with an increase in the injection current, an additional mechanism of hopping electrical transport with a variable jump length along localized states in the tail of the valence band arises, which leads to an increase in the conductivity of the films.

## 1. Introduction

As is known, amorphous carbon films have a dominant graphite-like structure, and therefore, they are also called graphite-like amorphous carbon films [1,2,3,4,5]. Such films are characterized by chemical inertness, high friction and wear characteristics [6,7,8], good conductivity, and biocompatibility [9]. Recently, graphite-like amorphous carbon films have attracted great interest due to their special properties such as single-component composition, p-type conductivity, and heterogeneity in the dielectric structure caused by conductive inclusions of graphite-like nanoclusters. It should be noted that due to the p-type conductivity, such films are promising for use as a hole transport layer in photovoltaic devices and solar cells [10].

The results of studying the structure of graphite-like amorphous carbon films are presented in detail in [11,12,13], where it was shown that the films consist of a mixture of two carbon fractions: diamond-like dielectric with sp^3^-type carbon hybridization and graphite-like conductive with sp^2^-type carbon hybridization. The graphite-like fraction forms sp^2^ nanoclusters, the sizes of which are no more than a few nanometers [14,15].

However, the electrical properties of carbon films with a predominance of the sp^2^ group have not yet been sufficiently studied, which hinders their application.

Measuring the I–V characteristics of graphite-like amorphous carbon films allows us to establish the influence of localized states on electrical conductivity. In turn, the analysis of temperature dependences of resistance allows us to determine the mechanisms responsible for the electrical transfer. This is due to the fact that localized states are closely related to the disordered structure of the carbon network and the presence of various defects in such films.

In this paper, we investigated the electrical properties of thin graphite-like amorphous carbon films with a non-uniform structure obtained by electron-beam evaporation in a vacuum and the effect of an electric field on the activation of charge carriers captured in localized states.

## 2. Materials and Methods

**Figure 1:**
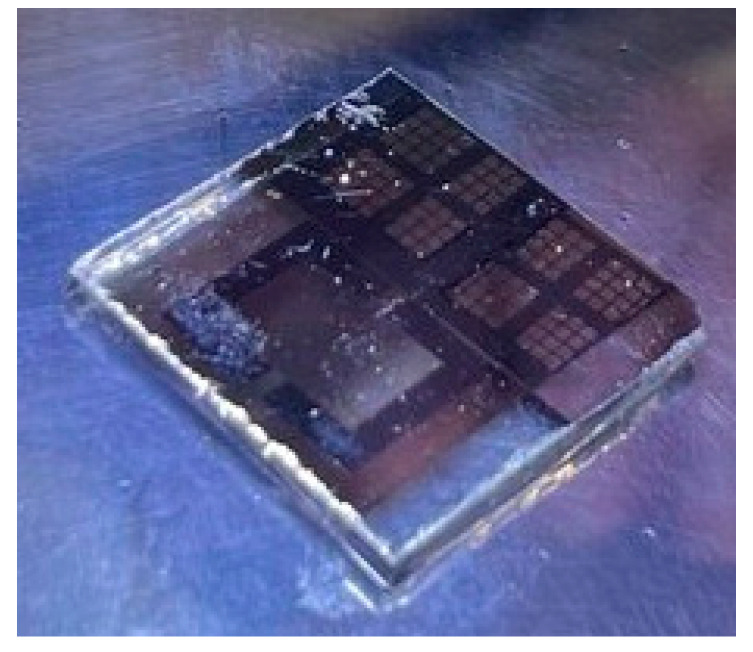
Photograph of the structure under study with formed contact pads for measurements.

Thin graphite-like amorphous carbon films (~50.0 nm) were deposited in vacuum on glass substrates with a 100 nm thick nickel layer and a silicon substrate (111) with a thermal SiO_2_ layer (for Raman spectroscopy) by electron-beam evaporation. After deposition, the films were annealed in vacuum at 973 K for 9 min (chamber pressure 5 × 10^−5^ Pa). Then, by electron-beam evaporation of nickel in vacuum through a mask, 100 nm thick upper metal contacts were formed to study the I–V characteristics and capacitance measurements. The area of the upper contact was 800 × 800 μm. The resulting structure is shown in Figure 1.

Transport and charge properties of the obtained structures were studied by measuring and analyzing the I–V characteristics (on a Progress-3000 characteristic curve recorder (NPK Progress, Moscow, Russia)) and C-V (on a TH512 capacitance–voltage analyzer (Tonghui Electronic, Changzhou, China)) using a SEMISHARE M6 probe station (SEMISHARE Technology, Shenzhen, China). During the measurements, the voltage on the studied structure varied in the range from −3.5 to +3.5 V. Before each measurement, the structure was kept under voltage for 1 min. This ensured more accurate and reliable current readings while measuring the I–V characteristics by the elimination of transient effects. To study the influence of the electric field effect, different bias voltages were applied to the samples during I–V characteristics’ measurement. It was assumed that in disordered carbon films, a change in the electric field leads to a change in the position of the Fermi level in the band of localized states. As a consequence, this affects the electrical transport of charge carriers.

Raman spectroscopy was applied as a simple and accurate method to identify different phases of carbon in the film. Due to its sensitivity to variations in translation symmetry, Raman spectroscopy allows distinguishing several types of carbon such as diamond, graphite, and diamond-like carbon. Raman spectra were recorded at room temperature using a scanning laser confocal micro-Raman spectrometer (Confotec NR500) (SOL instruments, Minsk, Belarus) with a 473 nm argon ion laser and a 600 lines/mm diffraction grating. Spectra were recorded in backscatter geometry using a 40× objective (NA = 0.75) with a 300 nm diameter laser beam focused on the sample plane. The signal accumulation time for each sample was 5 s.

## 3. Results and Discussion

**Figure 2:**
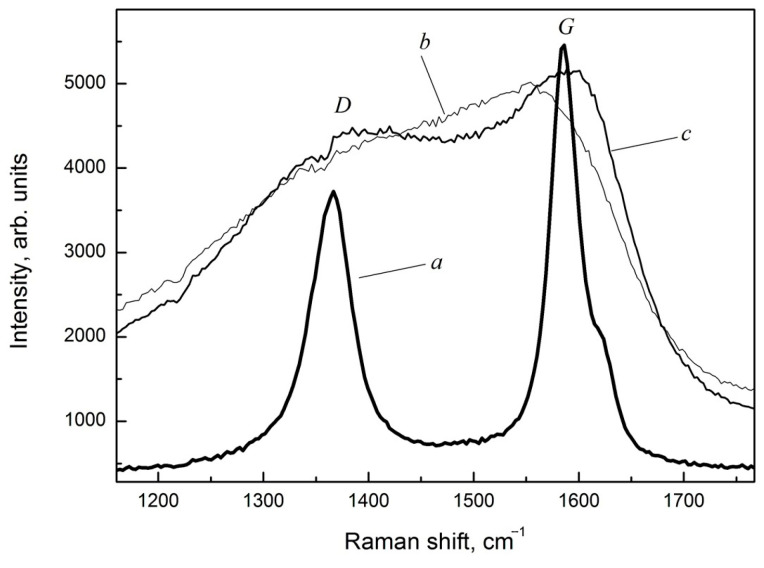
Raman spectra of the initial µc-graphite material (a) and graphite-like amorphous carbon films after deposition (b) and after heat treatment in vacuum (c).

Raman spectra of graphite-like amorphous carbon films and the initial µc-graphite material used as a target in electron-beam evaporation are shown in Figure 2.

**Figure 3:**
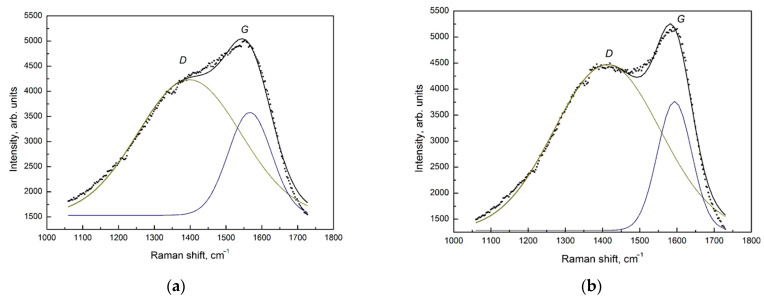
Raman spectra of graphite-like amorphous carbon films after deposition (**a**) and after heat treatment in vacuum (**b**) and their decomposition into two Gaussians.

The appearance of the carbon film spectra indicates a strong distortion and defectiveness of the structure of the graphite-like clusters. The microcrystalline graphite (µc-graphite) is characterized by the presence of a D band with a maximum of about 1352 cm^−1^ and a G line of about 1580 cm^−1^, which corresponds to the spectrum of the original microcrystalline graphite material consisting of very fine crystalline grains of graphite. The Gaussian decomposition of the Raman spectra of graphite-like amorphous carbon films into two peaks is shown in Figure 3.

Based on the decomposition presented in Figure 3, the main parameters of the D and G peaks, the intensity ratio of the *I_D_*/*I_G_* peaks, and the results of estimating the sizes of the graphite-like cluster regions were determined in accordance with [16] (Table 1).

From the analysis of the position of the G peak and the intensity ratio of the D and G bands (*I_D_*/*I_G_*) in accordance with [16], it was determined that the content of sp^3^ hybridized carbon in the films did not exceed 7%. This indicated that the obtained carbon films were graphite-like carbon films. The value of the G peak width at half maximum, equal to 91 cm^−1^ (full width at half maximum, FWHM) in accordance with [16], was characteristic of the size of the sp^2^ hybridized carbon regions of about 1.5 nm.

**Figure 4:**
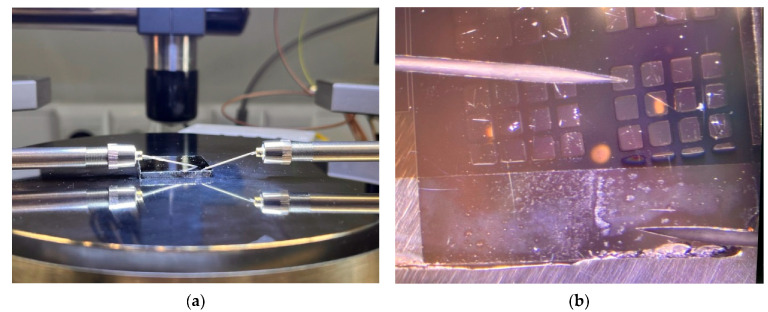
Photographs of the general view of the probe device in the working area (**a**) and the substrate with probes during the measurements of the I–V characteristics (**b**).

Measuring the I–V characteristics of the structures allowed us to analyze the nature of the change in the specific resistance ρ, to identify the effect of localized states in the forbidden band Eg, and to estimate their charge state, i.e., to determine the presence of traps, donors, or acceptors. Localized states are closely related to the structures of the carbon networks and the presence of various defects in them. Carbon defects may include vacancies, interstitial atoms, and dangling bonds. Conducting an analysis of the I–V characteristics allows obtaining information about localized states (traps) that can capture the injected charge carriers from the electrodes. C-V measurements were carried out in air at T = 298 K using a 4-wire circuit, which excluded the influence of contacts (Figure 4).

**Figure 5:**
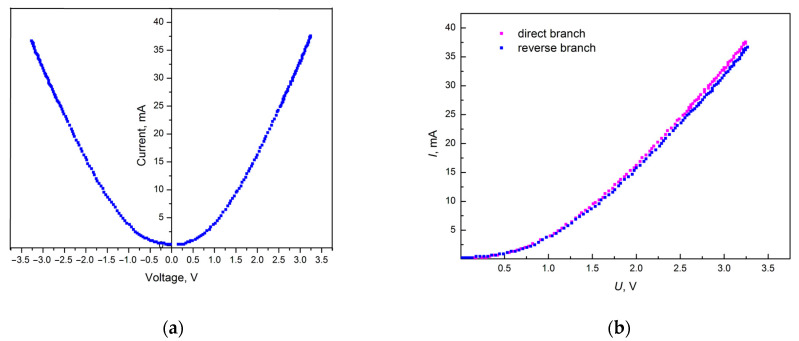
I–V characteristic of a graphite-like amorphous carbon film with positive and negative shear stress (**a**) and with the direct and reverse branches of the I–V characteristic combined (**b**).

Figure 5 shows the I–V characteristic of a graphite-like amorphous carbon film. The measurement results show that the characteristic is close to symmetrical (Figure 5a). Changing the sign of the voltage does not lead to a change in the nature of the curve (Figure 5b), which indicates the absence of contact influence.

**Figure 6:**
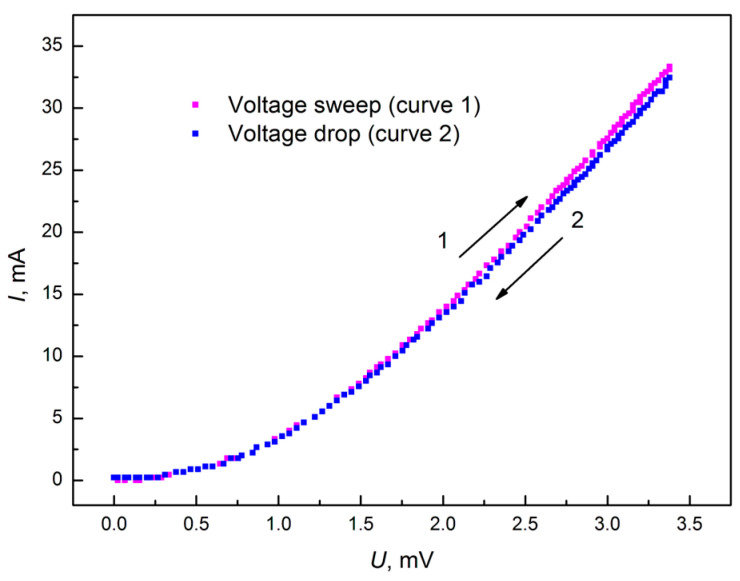
I–V characteristic of graphite-like carbon film during voltage sweep and voltage drop in the positive region.

Figure 6 shows the I–V characteristic of a graphite-like amorphous carbon film during voltage sweep and voltage drop. Curve 1 corresponds to the voltage sweep cycle. Curve 2 was taken during the voltage drop after the end of the sweep cycle. As can be seen from the obtained I–V characteristic curves, hysteresis was not observed. The absence of hysteresis indicates the stability of the electronic properties of such carbon films, which is important in applications such as microelectronics and sensors, where electrical polarization leads to undesirable effects.

**Figure 7:**
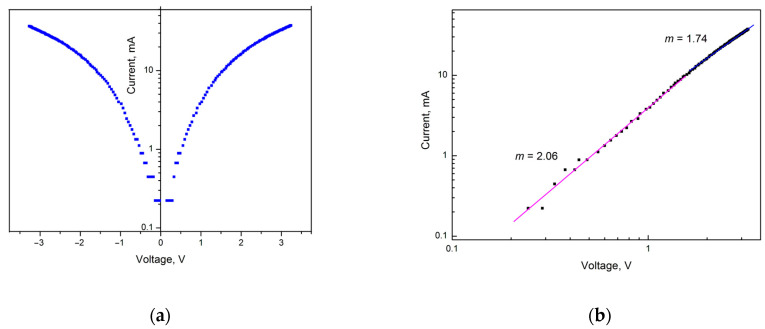
I–V characteristic of a graphite-like amorphous carbon film in semi-logarithmic (**a**) and logarithmic (**b**) coordinates.

To analyze the influence of localized states on charge transport in a graphite-like amorphous carbon film, it makes sense to present the I–V characteristic in semi-logarithmic and logarithmic coordinates (Figure 7).

The I–V curves in semi-logarithmic coordinates in Figure 7a demonstrate that the rate of current increase begins to decrease with increasing voltage. This behavior is caused by the space charge effect in the graphite-like amorphous carbon film. Logarithmic coordinates allow us to identify space charge limited currents (SCLCs). The representation of the I–V curve in logarithmic coordinates in Figure 7b shows the presence of two linear sections. The initial linear section has a slope of *m* = 2.06 (*R* = 0.99555), and the subsequent linear section has a smaller slope of *m* = 1.74 (*R =* 0.99936), which may indicate an increase in the rate of capture of injected charge carriers by traps due to the onset of a higher current flow.

When voltage is applied to the sample, some of the injected charge carriers are captured by traps. In the case of amorphous carbon films, which are characterized by p-type conductivity, the charge carriers are holes [17]. As is known, in the case of monoenergetic traps the I–V characteristic is quadratic; therefore, in logarithmic coordinates, this dependence gives a straight line with a slope equal to 2. This is exactly the result observed in the first linear section of the I–V characteristic with *m* = 2.06 for the level of deep monoenergetic traps. Monoenergetic traps have the same value of ionization energy—the energy of hole ejection from traps to the valence band. The slope tangent *m* = 1.74 of the second linear section shows that when a certain electric field strength (above 1700 mV) is reached, an additional mechanism of electrical transport appears in the graphite-like carbon film. As a consequence, the capture rate of injected charge carriers by traps increases.

**Figure 8:**
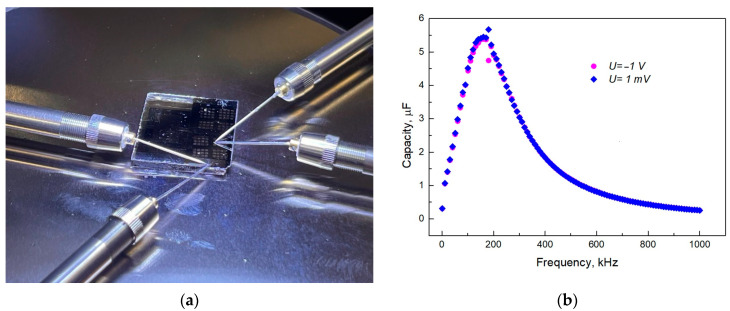
Photo of the working area of the probe device during C-V measurements (**a**) and the frequency response of the capacitance for structures with a semiconductor graphite-like carbon film at a negative −1.0 V and a positive shear voltage of 1.0 mV (**b**).

The frequency dependences of the capacitance of structures with a thin graphite-like carbon film, recorded by the 4-probe method (Figure 8a) at negative and positive shear stresses, are shown in Figure 8b.

As can be seen from Figure 8, the type of the frequency curve of the capacitance does not depend on the polarity of the applied shear stress. At low frequencies, for a thin graphite-like amorphous carbon film, a sharp increase in capacitance is observed, followed by a decrease in the high-frequency region. Such a course of the frequency dependence of the capacitance is associated with an increase in the permittivity at low frequencies and is also observed in [18] for C_60_ fullerite films at low frequencies. According to [19], an increase in the capacitance of dielectrics in the low-frequency region is associated with the Maxwell–Wagner polarization observed in inhomogeneous dielectrics with conducting inclusions. An increase in the real component of the permittivity in the low-frequency region occurs due to the accumulation of charges at the boundaries between the conducting and insulating fractions in such an inhomogeneous film. This is accompanied by partial screening of the external electric field, which is reflected as an apparent increase in the permittivity. As the frequency of the electric field increases, the accumulation of charges decreases, and the dielectric constant approaches its steady-state value.

In the case of graphite-like amorphous carbon films, graphite-like nanoclusters can act as conducting inclusions. For graphite-like amorphous carbon films, the main material is structural elements of nanometer sizes containing carbon in sp^2^- and sp^3^-hybrid states. These are conducting graphite-like clusters (sp^2^ hybridization) and their dielectric diamond-like shells (sp^3^ hybridization). The presence of graphite-like nanoclusters in carbon films was shown in [20] using transmission electron microscopy.

**Figure 9:**
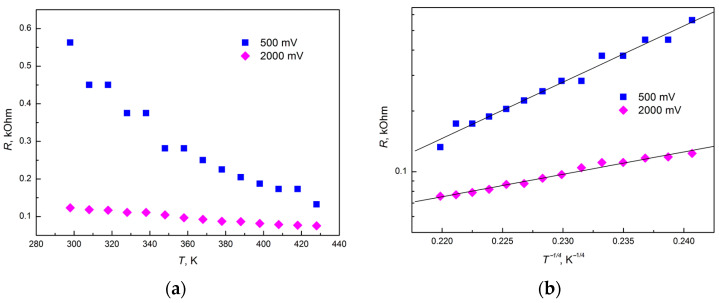
Change in resistance with temperature in normal (**a**) and semi-logarithmic coordinates depending on 1/T^1/4^ (**b**) for graphite-like amorphous carbon films in the temperature range of 298–428 K for bias voltages of 500 and 2000 mV.

To determine the mechanisms of electrical transport of thin graphite-like amorphous carbon films, the temperature dependences of resistance were studied in the temperature range of 298–428 K (Figure 9a) at bias voltages of 500 and 2000 mV (different electric field strengths). As can be seen from Figure 9, at a bias voltage of 2000 mV, a significant decrease in electrical resistance was observed. Analysis of the data in Figure 7b showed that in the temperature range of 298–428 K, the resistance in the coordinates lgR = f(1/T^1/4^) is well approximated by a straight line, which indicates a hopping mechanism of conductivity along localized states in accordance with Mott’s law [14,21,22]. It can be assumed that at a bias voltage of 500 mV in a graphite-like amorphous carbon film, a mechanism of electron conductivity with a variable hop length along localized states lying in a narrow energy band near the Fermi level is manifested, as shown in [17].

The sharp decrease in resistance when applying a bias voltage of 2000 mV can be explained by the occurrence of additional hopping electrical transport with a variable jump length over localized states in the tail of the valence band at temperatures close to room temperature, which was also observed in [17]. This is in good agreement with [23], where it was shown that more energy is required to activate charge carriers in the tails of the density of localized states than for states near the Fermi level.

## 4. Conclusions

The results of the I–V characteristics measurements of graphite-like amorphous carbon films showed that their characteristics are symmetrical. The change in the voltage sign did not lead to a change in the I–V characteristic, which indicates the absence of the influence of contacts. Hysteresis in the I–V curves obtained during the voltage sweep and decay was not observed. The I–V curve in logarithmic coordinates shows the presence of two linear sections. This is the initial linear section with a slope tangent of *m* = 2.06, which is typical for monoenergetic traps, and the subsequent linear section with a smaller slope tangent of *m* = 1.74, indicating an increase in the capture rate of injected charge carriers by traps due to the onset of a higher current flow.

For the frequency dependences of the capacitance of structures with a thin graphite-like amorphous carbon film, a sharp increase in capacitance is observed at low frequencies, followed by a decrease in the high-frequency region. The increase in capacitance in the low-frequency region is associated with the Maxwell–Wagner polarization, which is observed in inhomogeneous dielectrics with conductive inclusions. In the case of graphite-like amorphous carbon films, graphite-like nanoclusters act as conductive inclusions.

The results of the analysis of temperature resistance measurements show that at room temperatures, the mechanism of electron conductivity with a variable jump length along localized states lying in a narrow energy band near the Fermi level takes place. With an increase in the injection current and at a certain moment of filling the traps with charge carriers, an additional mechanism of hopping electrical transport with a variable jump length along localized states in the tail of the valence band also arises, which leads to an increase in the conductivity of the films.

## Figures and Tables

**Table 1:** Results of decomposition of Raman spectra of carbon films into Gaussians for peaks D and G and estimates of the sizes of regions of graphite-like clusters (l).

Film Type	Peak D, cm^−1^		Peak G, cm^−1^		*I_D_*/*I_G_*	l, nm
	ν	FWHM	ν	FWHM		
**After deposition**	1398	286	1567	121	1.18	0.2
**After annealing**	1412	285	1594	91	1.19	1.5

## Data Availability

The original contributions presented in this study are included in the article. Further inquiries can be directed to the corresponding author.
